# The Characteristics of* Staphylococcus aureus* Small Colony Variant Isolated from Chronic Mastitis at a Dairy Farm in Yunnan Province, China

**DOI:** 10.1155/2016/9157605

**Published:** 2016-03-15

**Authors:** Li-li Zhu, Feng-cai Zou, Yu-lin Yan, Qi-hui Wang, Yong-qiang Shi, Wei-jie Qu

**Affiliations:** Institute of Animal Science and Technology, Yunnan Agricultural University, Kunming 650051, China

## Abstract

*Staphylococcus aureus* is a major causative agent leading to bovine mastitis and has specific phonotypical characteristics including small colony, slow growth, and decreased hemolysis, therefore named as the small colony variants (SCVs). Out of 30 tested samples of the chronic* S. aureus* cases, one strain of SCVs (*S. aureus *SCV22) was isolated along with its parental strains (*S. aureus*11).* S. aureus *SCV22 showed a slow growth rate when it is compared with the parental strain. However, their resistant patterns were similar. Meanwhile,* S. aureus *SCV22 depicted the lower rate of apoptosis in bovine mammary epithelial cells. These findings of the present study presented the unique characteristics of* S. aureus *SCV22 for the first time in Yunnan province, which provided a prophase foundation for further study about the pathogenesis of* S. aureus* SCVs in chronic mastitis.

## 1. Introduction

Bovine mastitis is wildly prevalent among dairy cows mainly caused by the pathogenic* Staphylococcus aureus*, with a huge economic loss in dairy production. A hypothesis first proposed by Proctor et al. has showed that chronic or recurrent infection was associated with* S. aureus* SCVs [1]. Before the* S. aureus* SCVs were found,* S. aureus* was generally considered to be extracellular bacteria. However, many new reports showed that* S. aureus* SCVs owned the ability to persist intracellularly in nonprofessional phagocytes, such as epithelial cells, fibroblasts, osteoblasts, and endothelial cells [2, 3].

To the best of our knowledge, no reports have been documented for the isolation of* S. aureus* SCVs from dairy cows in Yunnan province, southwest China, as well as the related research. The aim of this study was to explore intracellular persistence, apoptosis of mammary epithelial cells, and establishment of mastitis model in* S. aureus* SCVs, and the results would provide prophase foundation to better know the infection mechanisms of* S. aureus* SCVs in chronic mastitis in further study.

## 2. Materials and Methods

### 2.1. Isolation and Identification of* S. aureus* and SCVs from Milk

Raw milk samples (*n* = 30) were aseptically gathered from scattered-feeding cows at a dairy farm in Kunming city of Yunnan province. Accordingly, all samples were cultured on Trypticase Soy Agar (TSA, HUANKAI, Guangzhou, China) complemented with 5% sheep blood (Ruite, Guangzhou, China) and then cultivated at 37°C for 24 h and 48 h, respectively.* S. aureus* was identified according to the phonotypical characteristics of being large, creamy, and forcefully hemolytic on TSA with 5% sheep blood. Potential SCVs colonies of tiny, nonpigmented, and nonhemolytic colonies on TSA with 5% sheep blood were also collected [4, 5]. Isolates of potential SCVs were subcultured on TSA for ten generations to assess their stability. SCVs and their parental strains were primarily tested for routine biochemical properties; then the potential SCVs were identified as* S. aureus* SCVs with the gene (nuc, nucA, and 16srDNA) by multiple PCR amplification (Table 1). The identified* S. aureus *SCV and its parental strains were termed as* S. aureus* SCV22 and* S. aureus*11, respectively. All isolates were stored at −80°C in Trypticase Soy Broth (TSB, HUANKAI) complemented with 25% volumes of glycerin (HUANKAI) for further investigation.

### 2.2. Auxotrophism Assay

Auxotrophy test of the SCVs was performed on Mueller-Hinton agar (MHA, Difco BD, Beijing, China) with 1 *μ*g/mL of hemin (Sigma, Shanghai, China), 1 *μ*g/mL of thymidine (Sigma), 1 *μ*g/mL of menadione sodium bisulfite (Sigma), and 100 *μ*g/mL of thiamine as described previously [6, 7]. Auxotrophism SCVs were identified, which showed the growth similar to their parental strains after incubating overnight at 37°C.

### 2.3. Bacterial Growth Curves

SCVs and their parental strains were analyzed in triplicate for growth curve features [1, 8]. The cryopreserved strains were inoculated into 10 mL TSB broth overnight at 37°C. 1 mL of overnight culture was transferred to a flask containing 200 mL TSB and then incubated at 37°C on a rotary shaker (150 rpm). Bacterial growth was determined by measuring the OD 600 nm per 4 hours.

### 2.4. Cell Culture

All reagents were purchased from Biological Industries (BI, Israel). The primary bovine mammary epithelial cells (Procell, Wuhan, China) were cultured in epidermal cell growth medium supplemented with 10% fetal calf serum (FCS, Sigma-Aldrich, Deisenhofen, Germany), epidermal growth factor (0.1 ng/mL), pig insulin, and hydrocortisone. The frozen cells were resuscitated in the cell culture plate with cell climbing piece in advance. The MECs were used after 3 passages for respective experiments.

### 2.5. Infection Experiments

For infection experiments [9], bacteria of* S. aureus* SCV22 and* S. aureus*11 were grown overnight at 37°C on TSA complemented with 5% sheep blood, picking a single colony to cultivate in liquid medium for 4 h, then collecting bacteria, suspending them with DMEM/F12 (sigma), and then adjusting the thalli concentration to 10^8^ cfu/mL. When the cells were grown to 80%, one cell was infected using 100 bacteria. The infection test was terminated after 3 h.

### 2.6. Apoptosis

The infected cells were dyed with Annexin V-FITC/PI kit (Beyotime, Shanghai, China) [10]. The amount of apoptosis cells was measured by flow cytometry (BD FACSCalibur, USA) and analyzed with Cellquest Pro software.

### 2.7. Scanning Electron Microscopy

The infected cells were washed 5 times with PBS and the cells were fixed with glutaraldehyde phosphate buffer. Then cell dehydration was performed for 15 min in each solution using a graded ethanol from 30% to 100%. The cell climbing was dried according to the critical point drying method before being sputter coated with gold in aE-1010 Ion Sputter Coater (Hitachi, Japan). Finally, the change of mammary epithelial cells was observed by A S-3000N scanning electron microscopy (Hitachi, Japan).

### 2.8. The Intracellular Persistence Assay

The infection experiment was conducted again and incubated for 3 h at 37°C with 5% CO_2_ to allow for the phagocytosis and adhesion of* S. aureus* SCV22 and* S. aureus*11. The tested cell plate was washed with sterile PBS and 100 mg/mL of gentamicin and 50 mg/mL of penicillin were added, respectively. The cell plate was washed again and lysostaphin was added to eliminate extracellular bacteria and then incubated for 30 min, 3 h, 9 h, and 12 h, respectively. At each time point, the cells were washed thrice with PBS to remove lysostaphin, which was followed by addition of 50 *μ*L 0.25% Triton X-100 to scatter epithelial cells and release intracellular bacteria. The cell lysing reagent was diluted with sterile water. 100 mL diluent was spread on the AGAR plate. The number of intracellular colony-forming units (CFU) was counted after* S. aureus *SCV22 and SASCV11 had grown overnight at 37°C at each time point.

### 2.9. Antimicrobial Susceptibility Testing

Antimicrobial susceptibility of* S. aureus *SCV22 was determined strictly with 10 antibiotics according to the drug susceptibility kit (Tianhe) instruction [11], while the standard* S. aureus* strain (ATCC29213, Tianhe, Hangzhou, China) was used as quality control (QC).

## 3. Results

### 3.1. Isolation and Identification of* S. aureus*11 and* S. aureus *SCV22 from Milk

**Figure 1:**
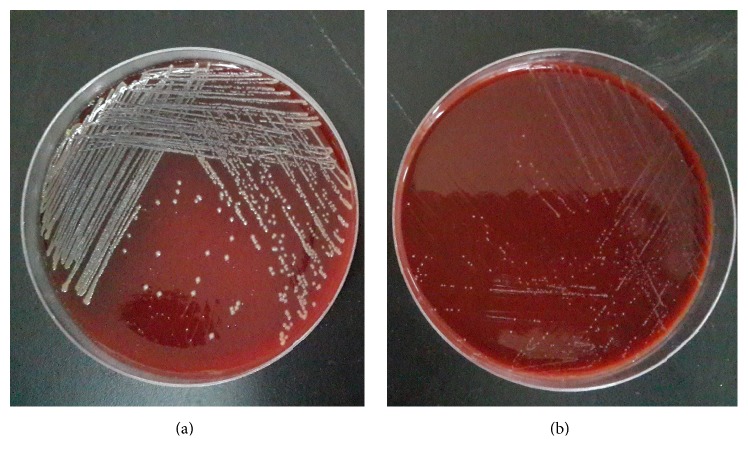
Colony morphological of* S. aureus *SCV22 with pinpoint colonies on Trypticase Soy Agar with 5% sheep blood (a) compared to* Staphylococcus aureus*11 (b).

Out of 30 samples collected from Holstein dairy cows, 8 isolates of suspected typical* S. aureus*11 were found. They were subcultured at 37°C for 16 h on TSA with 5% sheep blood. The result showed that all isolates have shown the typical morphology characteristics like (4 mm) *β*  hemolytic and creamy colonies (Figure 1(b)), and bacteria were Gram-positive coccus and arranged as grape-like clusters under light microscope. In addition, catalase test on pure TSA incubated at 37°C for 16 h was strong positive. Prevalence of coagulase was 100% by the commercial kit (Staphylase Test Kit, Oxoid, Beijing, China).

Out of eight* S. aureus* isolates, one was putative SCV22. Colonies of SCV11 with pinpoint (0.3 mm) were nonpigmented and failed to present an extremely slight hemolysis until incubated for 24 h at 37°C on TSA with 5% sheep blood (Figure 1(a)). Under the light microscope, these potential SCV11 appeared to be identical to typical* S. aureus*. For SCV11, catalase test was also positive, while coagulase was not detected. In the course of a continuous passage for ten generations these results were stable until the 10-generation passage.

**Figure 2:**
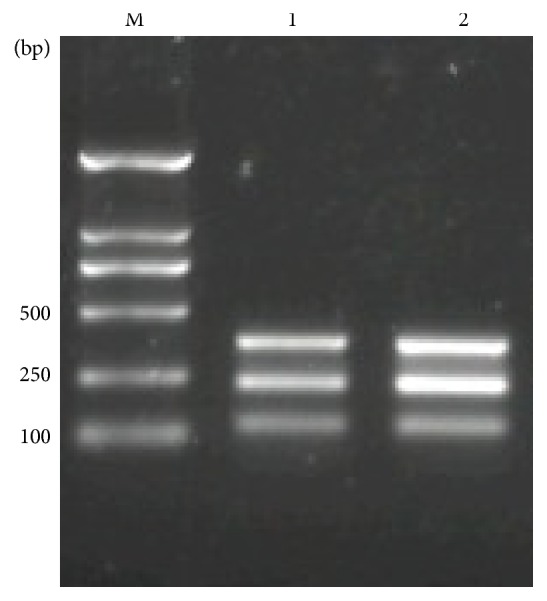
Molecular typing of* S. aureus*11 and* S. aureus *SCV22 isolated from cases of bovine mastitis by multiple Multiplex PCR. Molecular markers: 50 and 100 bp ladder (Takara, Japan).

All isolates were positive to multiple PCR test (Figure 2). The sequence analysis of 16S rRNA showed that gene of* S. aureus *SCV22 and its parental strains are homologous with published* S. aureus* sequence.

### 3.2. Auxotrophism Assay

**Figure 3:**
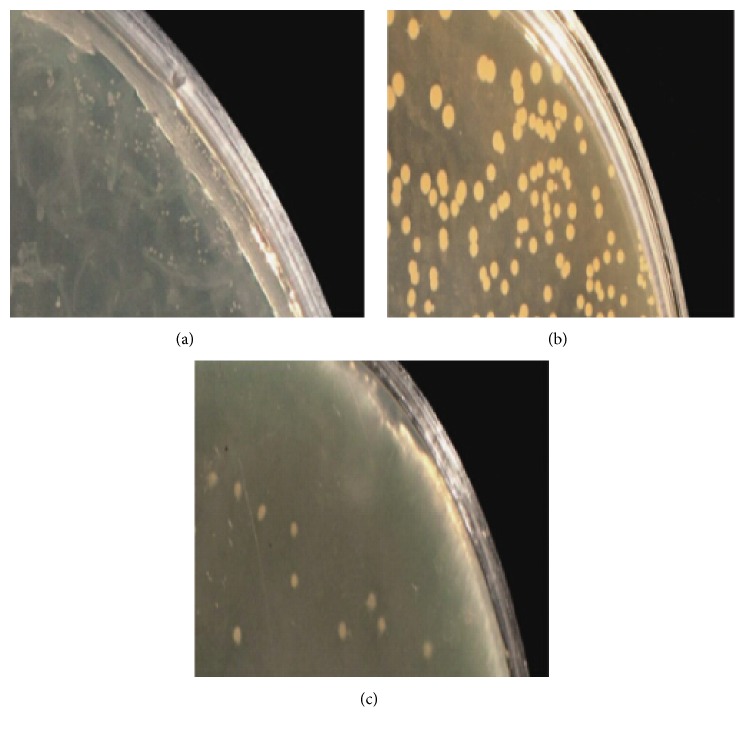
Auxotrophism assay: small colony variants* S. aureus* SCV22 (a), their parental strains (b), and auxotrophism strains (c).

The auxotrophism analysis of* S. aureus *SCV22 revealed a thymidine dependence; the auxotrophism strains were close to their parental strains on MHA with 1 *μ*g/mL of thymidine. However, the* S. aureus *SCV22 was negative for hemin, menadione, and thiamine (Figure 3).

### 3.3. Bacterial Growth Curves

**Figure 4:**
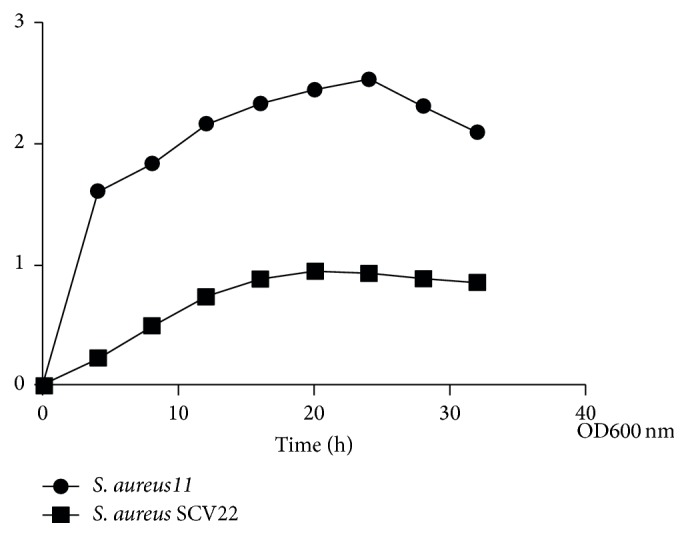
Growth curves of small colony variants* S. aureus *SCV22 in Trypticase Soy Broth compared to their parental strains.

According to the growth curves of* S. aureus *SCV22 and their parental strains (hereafter* S. aureus*11) cultured in pure TSB,* S. aureus*11 showed a typical bacterial growth curve. In contrast,* S. aureus *SCV22 showed a linear growth curve, which had an extended lag phase, but the boundary between the lag phase and log phase could not be distinguished because of none breakpoint shown in Figure 4.

### 3.4. Intracellular Assay

**Figure 5:**
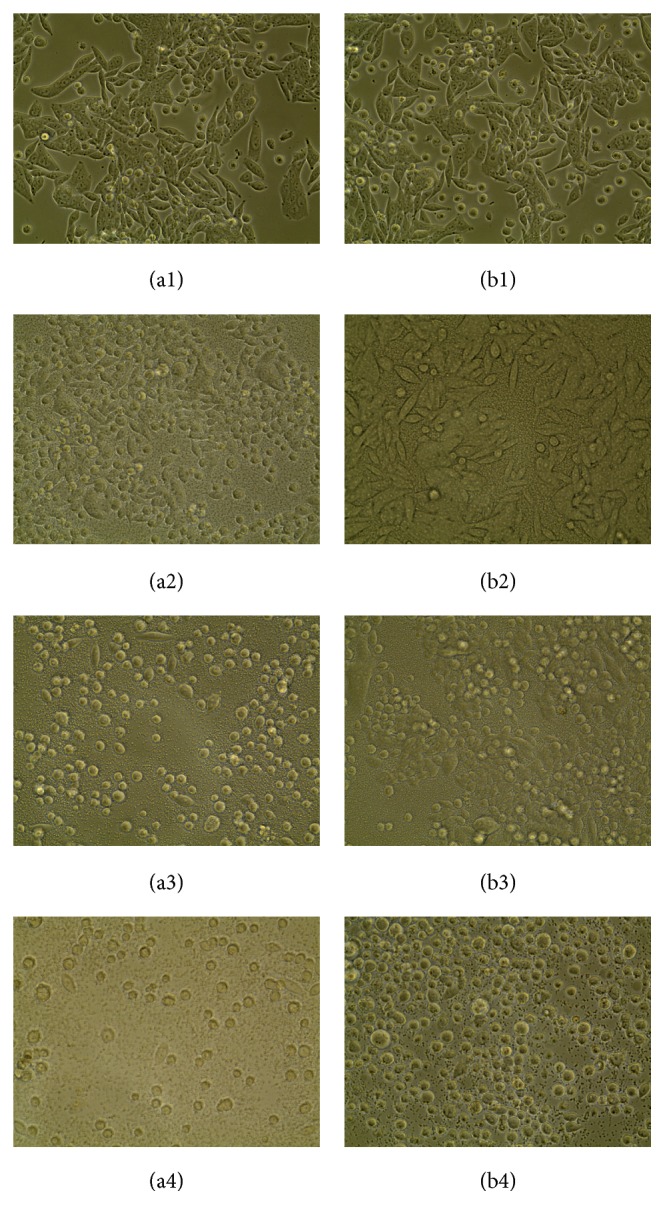
(40x) Morphological changes indicating cell damage, detachment, and rounding of primary bovine mammary epithelial cells infected with small colony variants* S. aureus *SCV22 and their parental strains after 0.5 hours (a1, b1), 3 hours (a2, b2), 9 hours (a3, b3), and 12 hours (a4, b4).* S. aureus*11 was presented in (a1), (a2), (a3), and (a4);* S. aureus *SCV22 was presented in (b1), (b2), (b3), and (B4).

**Figure 6:**
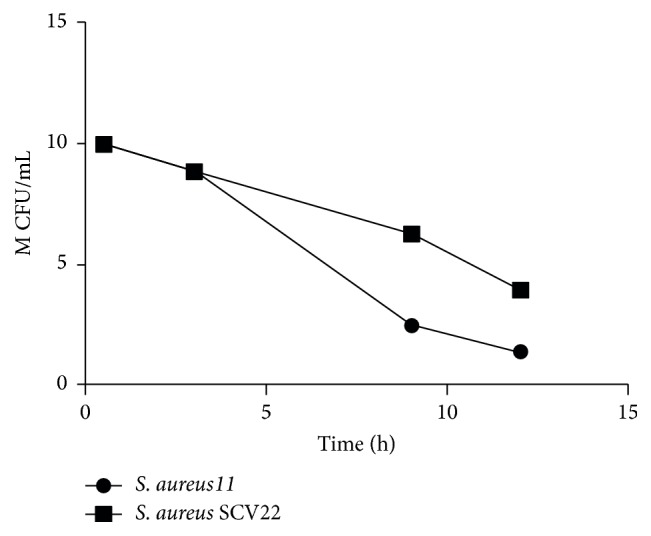
*S. aureus *SCV22 M CFU/mL (1 million colony-forming units/mL) recovered from primary bovine mammary epithelial cells at several time points compared to* S. aureus*11.

Assays for* S. aureus *SCV22 and* S. aureus*11 were done in triplicate to compare and quantify their ability to persist intracellularly within primary bovine mammary epithelial cells line. Measurement of CFU was conducted at the time points of 0.5 h, 3 h, 9 h, and 12 h, respectively (Figure 5). Hereby, prevalence of persistence from the time point of 3 h was becoming different between* S. aureus *SCV22 and* S. aureus*11, and the CFU number of* S. aureus *SCV22 was relatively stable (Figure 6).

### 3.5. Apoptosis

**Figure 7:**
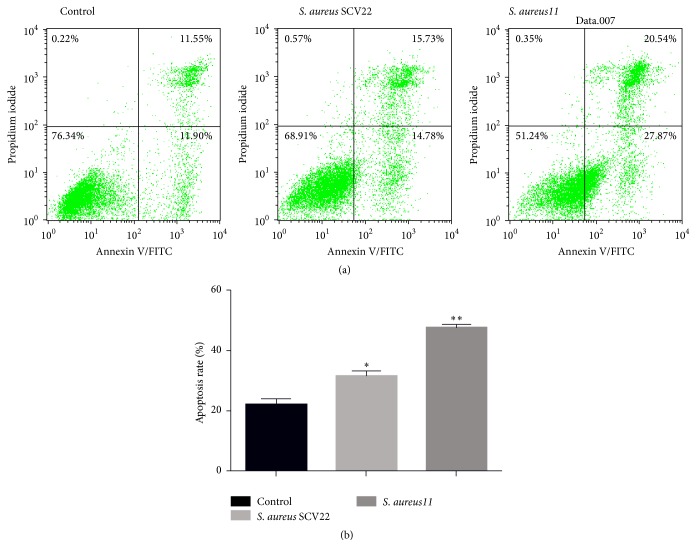
Small colony variants* S. aureus *SCV22 and their parental strain* S. aureus*11 induced apoptosis of primary bovine mammary epithelial cells (a, b). ^*∗*^There were significant differences between* S. aureus* SCV22 and control. ^*∗∗*^There is a very significant difference between* S. aureus*11 and control.

Compared to the control group, apoptosis rates of primary bovine mammary epithelial cells in infection groups of* S. aureus *SCV22 and* S. aureus*11 increased significantly (*P* < 0.05), and the difference between the* S. aureus *SCV22 groups and the* S. aureus*11 groups was also statistically significant (*P* < 0.01) (Figure 7).

### 3.6. Scanning Electron Microscopy

**Figure 8:**
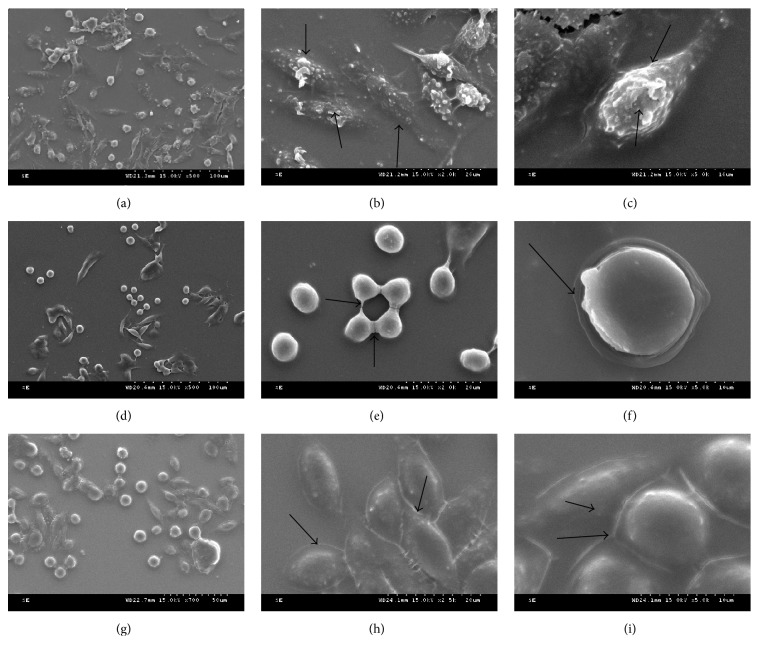
Representative scanning electron micrographs displayed the various effects of apoptosis (controls (g, h, and i), small colony variants* S. aureus *SCV22 (d, e, and f), and their parental strain* S. aureus*11 (a, b, and c), 5000x (c, f, and i), 2000x (b, e, and h), and 500x (a, d, and g)). Arrows in (b, c, e, f, h, and i) represented the morphology of cytomembrane.

Under the electron microscopy, control cells were grown normally. In the* S. aureus*11 infection groups, cells became round piece and flocked together after falling off, their cytomembrane ruptured, and the nucleus were unable to distinguish because of being shriveled. Cells of* S. aureus *SCV22 infection groups tended to flake, but cellular membrane was intact, and no extravasation of cytoplasm was found (Figure 8).

### 3.7. Antimicrobial Susceptibility Testing

The ATCC25923 was sensitive to 10 antibiotics. On the contrary, the* S. aureus *SCV22 had a resistance to six antibiotics; the same results were given in repeated test (Table 2).

## 4. Discussions

Bovine mastitis is wildly common among most of the cow farms caused by* S. aureus*, especially the chronic mastitis. Many reports showed that a small colony variant from* S. aureus* with special phonotypical characteristics was the major pathogenic bacteria leading to chronic mastitis [12–14]. In the present study, although* S. aureus *SCV22 and* S. aureus*11 had different phenotypic characteristics and growth curves, their targeted fragments were the same by multiple PCR amplification.

Since SCVs phenomenon was found from* Eberthella typhosa* strains by Jacobsen in 1910 [15], various SCVs from different bacteria have been reported [16–19]. Among them,* S. aureus *SCVs were wildly studied; their main characteristics are small colony, slow growth, forming biofilms, surviving in host cells chronically, and even reducing the sensitivity to antibiotics, which are consistent to our results, of which small phenotype and slow growth of SCVs are mainly caused by metabolic defects [10], and two types can be divided, including electron transfer defect and thymine synthesis defect. The former is the typical auxotroph; the normal phenotype can be formed by supplying menadione or hemin. In this study, the result showed that* S. aureus *SCV22 was thymine-dependent* S. aureus* SCVs; this type had been reported from patients with cystic fibrosis (CF) previously.

In the present study,* S. aureus *SCV22 had a high resistance to SXT, while their parental strains were sensitive. SXT can interfere with the synthesis of tetrahydrofolic acid to inhibit the growth of* S. aureus *[4]. In order to resist SXT, thymine-dependent SCVs absorbed the thymine in extracellular and survived ultimately. This phenomenon of thymine-dependent SCVs may be related to gene disruption mutation of thyA, because the thymidylic acid synthetase and tetrahydrofolic acid encoded by gene are important coenzyme for thymidylic acid synthetase.

In this study, the results of intracellular assay, apoptosis test, and scanning electron microscopy indicated that* S. aureus* SCVs persisted longer in nonprofessional phagocytes without profound damage, as previously reported [20–22]. This feature successfully avoided the host immunity and antibiotic therapy that partly explained its antibiotic resistance and the failure of antibody-mediated immune response (AMIR).

In conclusion, chronic mastitis is widespread in cow farms in Yunnan probability due to free-ranging, outdated production technology, lack of technical personnel, and abuse of antibiotics. In the study,* S. aureus* SCVs isolated from dairy cows were reported in Yunnan for the first time, which are strongly connected with chronic mastitis [23–25]. To date, there is no treatment to exhaustively eliminate chronic mastitis in production; therefore, further study should be carried out to better know the infection mechanisms of* S. aureus* SCVs in chronic mastitis.

## Figures and Tables

**Table 1:** Primer information.

Purpose gene	Sequence	Fragment length (bp)	*T*_*m*_
16SrDNA	Upstream primer: 5′-GGCGTTGCTCCGTCAGGCTT-3′	375	54°C
	Downstream primers: 5′-CGCTGGCGGCGTGCCTAAT-3′		
*nucA*	Upstream primer: 5′-CGCTTGCTATGATTGTGGTAGCC-3′	239	
	Downstream primers: 5′-TTCGGTTTCACCGTTTCTGGCG-3′		
*nuc*	Upstream primer: 5′-TCGTCAAGGCTTGGCTAAAGTTGC-3′	126	
	Downstream primers: 5′-TCAGCGTTGTCTTCGCTCCAAA-3′		

**Table 2:** Susceptibility test of ATCC 25923 and *S. aureus* SCV22 isolates.

Antibiotic	ATCC 25923	*S. aureus *SCV22
Fosfomycin	S	S
SXT	S	R
Oxacillin	S	S
Ampicillin	S	R
Vancomycin	S	R
Streptomycin	S	R
Vancomycin	S	S
Ampicillin	S	R
Oxacillin	S	S
Gentamicin	S	R

S: sensitive; R: resistant.

SXT: sulfamethoxazole trimethoprim.
